# Targeting ferroptosis regulators in lung cancer: Exploring natural products

**DOI:** 10.1016/j.heliyon.2024.e33934

**Published:** 2024-07-02

**Authors:** Wang Yuhao, Cheng Shenghua, Chen Jueying, Xiang Shate, Song Rongrong, Shen Xiangfeng

**Affiliations:** a Graduated College, Jiangxi University of Chinese Medicine, Nanchang, 330000, Jiangxi, China; b First Clinical Medical College, Zhejiang Chinese Medicine University, Hangzhou, 310053, Zhejiang, China; c Department of Nephrology, Jinhua Hospital of Traditional Chinese Medicine, Jinhua, 321017, Zhejiang, China

## Abstract

Lung cancer remains a formidable global health challenge, necessitating innovative therapeutic strategies for improved efficacy. This review explores the untapped potential of natural products and Traditional Chinese Medicine (TCM) in lung cancer therapy, focusing on targeting ferroptosis regulators. Natural compounds, such as curcumin and resveratrol, exhibit diverse anti-cancer mechanisms, complemented by TCM's holistic approach rooted in a 3500-year history. Emphasizing the induction of cell death, particularly ferroptosis, the review highlights its significance in overcoming challenges like resistance to conventional therapies. Key ferroptosis regulators are explored in the context of natural products and TCM. The impact of these treatments on crucial pathways, such as antioxidant mechanisms (GPX4, SLC7A11, and NRF2), iron metabolism regulators, and lipid and mitochondria pathways, is examined. The findings provide a comprehensive overview of how natural products and TCM modulate ferroptosis in lung cancer, offering valuable insights for the development of innovative, side-effect-reduced therapeutic strategies. This work holds promise for transforming the landscape of lung cancer treatment by integrating the rich resources of nature into conventional therapeutic paradigms.

## 1 Introduction

Lung cancer poses a significant worldwide health challenge, standing as the second most frequently diagnosed cancer and the primary cause of cancer-related fatalities in 2020. Accounting for 2.2 million new cases and 1.8 million deaths, it comprises 11.4 % of all cancer diagnoses and 18.0 % of cancer-related deaths. Despite progress in screening techniques, the 5-year survival rate remains modest, ranging from 10 % to 20 %. Ongoing emphasis on prevention and early detection is imperative [1,2]. The management of lung cancer faces difficulties, encompassing enduring unfavorable prognoses, the presence of challenging rare tumors without efficient treatments, and constraints in diagnosing peripheral lung cancers. Overcoming resistance to targeted therapies, like osimertinib, presents obstacles, and there is a need for deeper exploration into the intricacies of immunotherapy responses and the identification of predictive biomarkers [3,4]. Natural products offer valuable contributions in combating lung cancer through diverse mechanisms. Compounds like curcumin and resveratrol exert anti-cancer effects by inducing apoptosis, halting cell cycle progression, inhibiting angiogenesis, and generating reactive oxygen species (ROS). These substances also showcase anti-inflammatory properties, induce modifications at the epigenetic level, and overcome drug resistance when combined with traditional chemotherapy. Moreover, specific compounds target signaling pathways, disrupt pathways related to resistance, and enhance the effectiveness of standard treatments. Natural products demonstrate potential in immunomodulation, prompting cellular senescence, minimizing adverse effects, and potentially restricting metastasis. As research advances, the incorporation of natural products into lung cancer treatment may introduce innovative therapeutic strategies with reduced side effects [5,6]. Rooted in a 3500-year history, Traditional Chinese Medicine (TCM) functions as a healthcare system aimed at reinstating bodily balance and harmony. TCM encompasses two primary categories: TCM materials, involving herbal and natural elements, and TCM preparations, encompassing diverse formulations for specific conditions. Although historically underexplored, TCM's effectiveness is now a subject of scientific investigation. In lung cancer treatment, TCM serves as adjuvant or maintenance therapy, potentially reducing side effects and enhancing overall survival. The approach embraces a holistic perspective on health, emphasizing equilibrium in vital energies and blood circulation. TCM's utilization of natural products aligns with its traditional principles, contributing to its therapeutic mechanisms [[7], [8], [9], [10]]. The induction of cell death by natural products is crucial in lung cancer therapy due to its potential to selectively eliminate cancer cells while minimizing harm to normal cells. Natural products often possess bioactive compounds that can target specific pathways involved in cancer cell survival and proliferation. Apoptosis, a regulated and controlled form of cell death, is a key mechanism through which these natural products induce the demise of cancer cells [11,12]. Ferroptosis is a unique type of cell death dependent on iron, marked by a disturbance in cellular redox balance that results in heightened reactive oxygen species (ROS) levels. Coined by Dixon et al., this form of cell death is triggered by erastin and stands apart from conventional cell death mechanisms [13]. Its association with degenerative diseases, ischemic conditions, and cancer development implicates ferroptosis in various pathological processes. Enzymes such as glutathione peroxidase 4 (GPX4) play a crucial role in regulating ferroptosis, and it has emerged as a potential target for cancer therapy. Additionally, ferroptosis has been linked to resistance in cancer treatments, making it a focal point for understanding and addressing challenges in therapeutic interventions [14]. Ferroptosis holds significance in lung cancer therapy as an innovative and promising strategy to address the limitations associated with conventional treatments. Cancer cells, including those in lung cancer, exhibit a heightened dependence on iron, rendering them susceptible to ferroptosis. Inducing ferroptosis in lung tumors emerges as a means to hinder the progression of lung cancer and mitigate resistance to established therapies. The distinctive features of ferroptosis, particularly its reliance on iron metabolism, set it apart from other programmed cell death pathways, offering a potential breakthrough in overcoming challenges in lung cancer treatment [15,16]. Ferroptosis-related signaling pathways and regulators have been widely discovered in lung cancer in recent reviews [15,17,18]. However, the impact of natural products and, especially traditional Chinese medicine has not been thoroughly explored on the ferroptosis regulators. Therefore, this review aims to provide the current standpoint of targeting ferroptosis regulators by these compounds in lung cancer for the first time.

## 2 Antioxidant pathways as a target of natural products

**Fig. 1:**
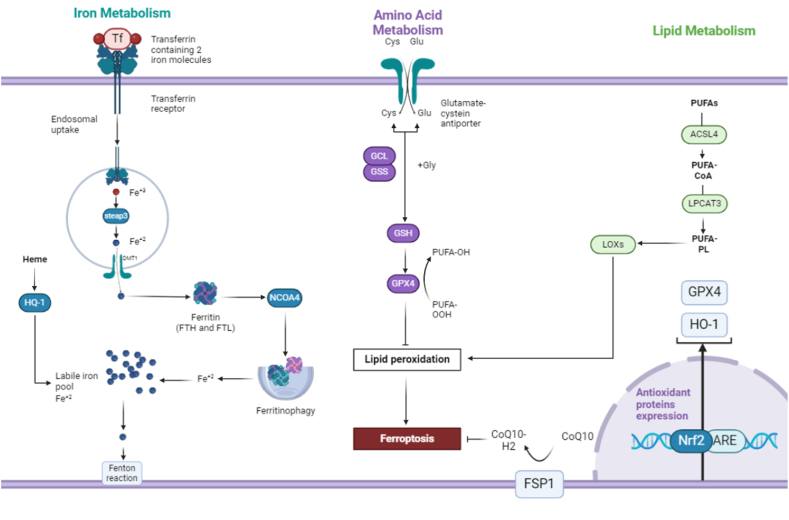
Ferroptosis is an iron-dependent form of regulated cell death characterized by the accumulation of lipid peroxides. The process is initiated by the depletion of glutathione (GSH) or inhibition of glutathione peroxidase 4 (GPX4), leading to the accumulation of reactive oxygen species (ROS) and lipid peroxidation. Iron catalyzes the formation of highly reactive hydroxyl radicals through the Fenton reaction, further exacerbating lipid peroxidation and membrane damage. Key regulators and pathways involved include the cystine/glutamate antiporter system (System Xc-), the mevalonate pathway, and various antioxidant defenses. The figure highlights the interplay between these components, showcasing the crucial steps and molecular players in ferroptosis.

Ferroptosis, a programmed cell death variant, encompasses intricate pathways pivotal for its initiation. The anti-oxidant pathway revolves around GPX4, a critical enzyme in reducing phospholipid hydroperoxides. Blocking GPX4 results in the buildup of these hydroperoxides, leading to cell membrane impairment and ferroptotic demise. The iron metabolism pathway heightens susceptibility to ferroptosis by regulating the labile iron pool (LIP), which is essential for generating reactive oxygen species. Precise control of cellular iron levels is disrupted during ferroptosis induction. The lipid metabolism pathway is characterized by increased lipid peroxidation, influenced by both nonenzymatic and enzymatic processes. Grasping these pathways offers insights into ferroptosis initiation, presenting potential therapeutic avenues, especially in overcoming chemotherapy resistance in cancer treatment [14]. The antioxidant pathway in ferroptosis is intricately linked to the activation of key components such as system Xc^−^, GPX4, and NRF2, particularly in the context of cancer. System Xc^−^, responsible for cystine import, plays a pivotal role by providing the necessary substrate for glutathione synthesis. GPX4 serves as a critical enzyme in counteracting lipid peroxidation, a hallmark of ferroptotic cell death. Additionally, the transcription factor NRF2 orchestrates the cellular defense against oxidative stress by regulating the expression of antioxidant genes. In the context of cancer, the dysregulation of these components can contribute to the vulnerability of cancer cells to ferroptosis, making them potential targets for therapeutic interventions aimed at exploiting this unique cell death pathway (Fig. 1) [19].

### 2.1 System Xc-

System Xc^−^ stands as a crucial element in the ferroptotic pathway, serving as the pivotal upstream node in the System Xc^−^/GSH/GPX4 axis. Comprising the catalytic subunit xCT (SLC7A11) and regulatory subunit 4F2hc (SLC3A2), connected by disulfide bonds, this antiporter operates as a chloride-dependent and sodium-independent transporter of cysteine (Cys) and glutamate (Glu), facilitating their exchange in a balanced ratio. Activation of SLC7A11 expression plays a vital role in reinstating redox homeostasis and supporting cell survival during diverse stress conditions, encompassing oxidative stress, amino acid scarcity, metabolic stress, and genotoxic stress. Cys acquired through System Xc^−^ is crucial for glutathione (GSH) biosynthesis, a key antioxidant in mammalian cells. Disruptions in intracellular Cys and GSH levels directly impact the activity of GPX4, potentially triggering ferroptosis. Compounds like Erastin and its counterparts interfere with System Xc^−^, inducing effects such as cysteine scarcity, GSH depletion, endoplasmic reticulum stress, and eventual cell death. While intracellular Cys can originate from alternative sources, malfunction in System Xc^−^ results in Cys scarcity and GSH depletion, heightening cellular vulnerability to ferroptosis. Targeting System Xc^−^ emerges as a promising avenue for inducing ferroptosis and addressing drug-resistant solid tumors in cancer treatment [20]. SLC7A11 is under the control of many oncogenes (e.g., MiR-27a-3p, RBMS1, SOX2) in lung cancer, leading to its overactivity and ferroptosis induction resistance [[21], [22], [23]]. Many natural products that target SLC7A11 in lung cancer have been discovered (Table 1). In addition to SLC7A11, some of these compounds can induce ferroptosis via altering the expression of other ferroptosis regulators, particularly GPX4 [[24], [25], [26], [27], [28], [29]]. The tumor growth inhibitory function of natural SLC7A11 inhibitors in lung cancer has been observed in vivo as well, making them as possible candidate for future studies in clinical settings. Compounds like β-elemene can be used as combined therapy with chemotherapy agents like Erlotinib to improve the treatment efficacy by targeting SLC7A11 and downregulating its expression [30].

**Table 1:** Natural products targeting pathways of the ferroptosis in lung cancer.

Natural product name	Source	Dose	Cell Lines	Other Targets	Effects	Model
Amino acid metabolism						
SLC7A11						
Resveratrol [62]	Grapes	5–100 μmol/L	H520	GPX4, ACSL4, TFRC, and HMMR	Improved CD8 +T cells function ↓ Cell viability	In Vitro
Juglone [24]	Walnut family (Juglandaceae)	10–50 μM	A549	MDA, GPX4, NRF2	↑Concentration of Fe^2+^ and ROS ↓ Colony Formation	In Vitro
Gigantol [25]	Orchid species within the Dendrobium genus	50, 100, and 150 μM	H460 and A549	GPX4	↑ROS levels and lipid peroxidation ↓ Tumor volume and weight	In Vivo and In Vitro
β-elemene [30]	Curcuma wenyujin	100 mg/kg (In vivo)	EGFR-mutant (H1975, H1650, and H1819)	LncRNA H19/GPX4/	Combined with Erlotinib can decrease cell viability, induce ROS, reduce tumor volume and promote ferroptosis	In Vivo and In Vitro
Andrographolide [26]	Andrographis paniculate	10 mg/kg	H460 and H1650	GPX4	↓Proliferation, migration, ↑mitochondria dysfunction, and ↓tumor growth and metastases	In Vivo and In Vitro
Valtrate [27]	V. jatamansi Jones	1.25–40 μM	A549 and H1299	GPX4	↓ Proliferation and tumor growth	In Vivo and In Vitro
Capsaicin [28]	Chili pepper	50–300 μM	A549 and NCI–H23 cells	GPX4	↓ Cell viability	In Vitro
Solasonine [29]	Solanum plants	10–30 μM	Calu-1 and A549	GPX4	↓ Growth ↑ Mitochondrial Injury	In Vitro
Dihydroartemisinin [63]	Artemisia annua	20–60 μM	NCI–H23 and XWLC-05	PRIM2/β-Catenin	↓ Proliferation, colony formation ↓Tumor growth	In Vivo and In Vitro
Sulforaphane [64]	Cruciferous vegetables	20 μM	H69, H82 and H69AR	–	↓ Growth ↑Intracellular levels of Fe^2+^	In Vitro
GPX4						
β-Elemene [32]	Curcuma wenyujin	3.125–50 μM	A549, NCI–H460 and SPC-A-1	TFEB	↓Tumor growth ↑lipid ROS	In Vivo and In Vitro
Timosaponin AIII [33]	Anemarrhena Asphodeloides	4 μM	H1299, A549, SPC-A1	HSP90	↓ Cell proliferation and migration ↑ Cell cycle arrest	In Vivo and In Vitro
Melittin [65]	Bee venom	–	A549	CHOP	↑ ROS burst	In Vitro
Dihydroartemisinin [34]	Artemisia annua	30–60 μM	Lewis cells	NF-κB TfR1 COX-2	Exhibited apparent M1 phenotype reprogramming	In Vivo and In Vitro
Red ginseng polysaccharide [66]	Red ginseng	50-1600 μM	A549	–	↓ Cell proliferation and migration	In Vitro
Fuzheng Kang'ai Decoction [35]	12 CHMs	Variable	A549, PC9, H1975, H1650, and H1299	SLC7A11	↓ Growth	In Vivo and In Vitro
Dihydroartemisinin [36]	Artemisia annua	5–100 μM	Lewis cells	TfR1- COX-2	DHA and Ce6 PDT reduced GPX4 expression and increased ROS, leading to drug sensetivity	In Vitro
Bufotalin [67]	Venenum bufonis	1–4 μM	A549	–	↓ Cell proliferation GPX4 ubiquitination and degradation	In Vivo and In Vitro
Dihydroisotanshinone I [68]	Danshen	10–30 μM	A549 and H460	–	↓ Cell growth and	In Vivo and In Vitro
NRF2						
Eriocitrin [69]	Lemon and citrate juice	50 μM	A549 and H1299	SLC7A11, GPX4 and FTH1	↓Proliferation, migration, and EMT	In Vitro
Isoorientin [54]	Passion Fruit	–	A549	SIRT6/GPX4	↓ Cell growth, colony formation, and tumor volume	In Vivo and In Vitro
Qingrehuoxue Formula [55]	Decoction	15 g/kg	A549	GSK-3β/SLC7A11 and GPX4	↓ Tumor volumes ↓Proliferation ↓Angiogenesis and metastasis	In Vivo and In Vitro
Manoalide [52]	Sponges	15 μM	A549, H157, HCC827, and PC9	SLC7A11/FTH1	↓ Proliferation ↑ER vacuolation ↑ Mitochondrial ROS ↓EGFR-TKI resistance combined with osimertinib	In Vitro
Trabectedin [70]	Ecteinascidia turbinate	2–20 μM	A549, H460, PC-9, H1299	TFR1/GPX4/HIF-1α	↓ Cell viability ↑Iron-dependent accumulation of ROS	In Vitro
Ginkgetin [53]	Ginkgo biloba	5 μM	A549	SLC7A11/GPX4/HO-1/TF	↑ Cisplatin-induced cytotoxicity ↓ Tumor weight and cell viability	In Vivo and In Vitro
Brusatol [51]	Brucea javanica	0.5 mg/kg	A549, H838 and H1703	FOCAD-FAK/SLC7A11/GPX4	↓ Tumor growth and cell viability	In Vivo and In Vitro
FSP1						
Curcumin [39]	Curcuma Longa	10–80 μmol/L	A549	GPX4	↓ Cell viability ↓Tumor growth	In Vivo and In Vitro
Transcription Factors						
STAT3						
Zerumbone [57]	Zingiber zerumbet Smith	6.25–100 μM	A549 and H460	AKT/STAT3/SLC7A11	↓Proliferation and migration	In Vitro
Artemisia santolinifolia (AS) [58]	“wormwood” semi-shrub	100 and 200 μg/mL	A549 and H23	GPX4	↑Chemosensitization ↓Proliferation	In Vitro
EGCG [59]	Green tea	20 μM	A549	SLC7A11	↓ Tumor growth and cell viability ↓Proliferation, migration, and invasion	In Vivo and in vitro
Fascaplysin [60]	Sponges	0.5–2 μM	A549	SLC7A11/GPX4	↓Proliferation and migration ↓ Tumor growth and cell viability	In Vivo and In Vitro
Shikonin [61]	Lithospermum erythrorhizon (Zicao)	0.5–5 μM	SBC-2 and H69 (SCLC)	GPX4/SLC7A11	↓Proliferation, migration, and invasion ↓ Tumor growth	In Vivo and In Vitro
Natural product name	Source	Dose	Cell Lines	Other Targets	Effects	Model
Iron Metabolism						
TFRC						
Trabectedin [70]	Ecteinascidia turbinate	2–20 μM	A549, H460, PC-9, H1299	Nrf2/GPX4	↓ Cell viability	In Vitro
Sinapine [71]	Rapeseed and cruciferous plant	2–10 μM	A549, SK, and H661	SLC7A11/GPX4	↓ Cell proliferation ↓Tumor growth	In Vivo and In Vitro
DMT1						
HO-3867 (Curcumin Analog) [72]	Curcuma longa L	5–80 μM	H460, PC-9, H1975, A549, and H1299	SLC7A11/GPX4	↓ Cell viability	In Vitro
FPN						
Sclareol [73]	Salvia sclarea	12.5–100 μM	A549	GPX4/TFR1/FTh	↓Proliferation, colony formation, cell migration, and invasion	In Vitro
FTH						
Dihydroartemisinin [63]	Artemisia annua	12.5–50 μM	A549-GR	GPX4	↓Proliferation and gefitnib resistance	In Vitro
Curcumenol [74]	Curcuma wenyujin	200–300 μg/ml	H1299 and H460	lncRNA H19/miR-19b-3p/FTH1	↓ Cell proliferation ↓Tumor growth	In Vivo and In Vitro
NCOA4						
6-Gingerol [75]	Ginger	20–80 μM	A549	FTH1, GPX4 and ATF4	↓ Cell proliferation ↓Tumor growth	In Vivo and In Vitro
Pseudolaric acid B [76]	Pseudolarix kaempferi	1–2 μM	A549	FTH1	↓Proliferation and migration	In Vitro
d-Borneol [77]	Cinnamomum cam phora	0.5–4 μg/ml	H460	HO-1	↓ Cell proliferation ↓Tumor growth ↑Cisplatin sensitivity	In Vivo and In Vitro
Curcumin [78]	Curcuma wenyujin	30 μM	A549 and H1299	ACSL4/SLC7A11/GPX4	↓ Cell proliferation ↓Tumor growth	In Vivo and In Vitro
Lipid Metabolism						
ACSL4						
Scoparone [79]	Artemisia capillaris Thunb	100 μM	A549, H1299 and PC-9	SP1	↓Proliferation, cell migration, and invasion	In Vitro
Mitochondria						
VDACs						
Hedyotis diffusa injection [80]	Hedyotis diffusa	10–60 μg/ml	A549 and H1975	–	↓Proliferation, cell migration, and invasion ↓Tumor growth	In Vivo and In Vitro
Mitochondrial Ca^2+^						
Erianin [81]	Dendrobium chrysotoxum Lindl	12.5–100 nM	H460 and H1299	GPX4, SLC40A1,SLC7A11	↓Proliferation, cell migration, and invasion ↓Tumor growth	In Vivo and In Vitro
Diplacone [82]	Paulownia tomentosa Mature Fruit	1.25–40 μM	A549	–	↓ Cell viability	In Vitro

### 2.2 GPX4

**Fig. 2:**
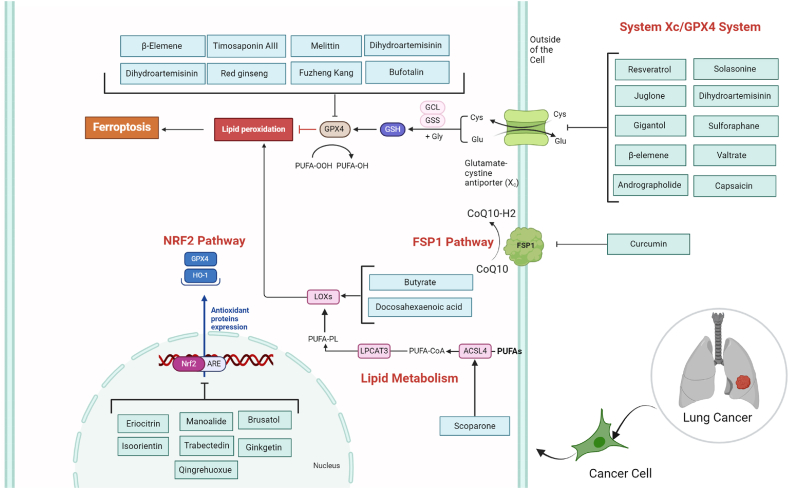
Deciphering the role of natural products in tumor suppression of lung cancer by affecting anti-oxidant pathways (System Xc^−^/GPX4, NRF2, and GPX4) and lipid metabolism pathway and ferroptosis induction.

GPX4 plays a crucial role in the regulation of ferroptosis and system Xc^−^ facilitates the uptake of cystine, contributing to GSH biosynthesis. The availability of GSH, in turn, is vital for the enzymatic activity of GPX4, which acts as a key regulator in preventing ferroptotic cell death by counteracting lipid peroxidation. The collaboration between system Xc^−^ and GPX4 is integral to the cellular defense against ferroptosis. GPX4 is identified as the key upstream regulator in the ferroptotic process. Its unique function lies in reducing complex hydroperoxides, including phospholipid hydroperoxides, thus interrupting the lipid peroxidation chain reaction. This activity of GPX4 is essential for preventing ferroptotic cell death. The availability of cysteine, biosynthesis of GSH, and the proper functioning of GPX4 are highlighted as central components in maintaining resistance to ferroptosis [31]. GPX4 is upregulated in many patients with lung cancers and causes resistance to epidermal growth factor receptor-tyrosine kinase inhibitors (EGFR-TKIs), radiotherapy, and immune checkpoint inhibitors [15]. The majority of the studies discussing the inhibitory effects of natural products in lung cancer introduced GPX4 as a promising target that can be downregulated (Table 1). Many natural products can cause GPX4 degradation in lung cancer and induce ferroptosis. β-ELE, a compound under investigation for its anti lung cancer properties in clinical settings across China, demonstrates a binding affinity to TFEB, a master regulator of lysosome biogenesis. This interaction activates TFEB, leading to increased lysosomal biogenesis and the degradation of GPX4, a negative regulator of ferroptosis, through a lysosome-dependent mechanism. The reduction in GPX4 results in enhanced lipid ROS levels and elevated intracellular iron, indicative of ferroptosis induction. In an in vivo setting, β-ELE demonstrates significant tumor growth inhibition, an effect compromised in the absence of TFEB, reinforcing the crucial role of TFEB in mediating β-ELE-induced anticancer responses in lung cancer [32]. Through a similar mechanism, Timosaponin AIII (Tim-AIII) exerts its anticancer effects in non-small cell lung cancer (NSCLC) by inducing ferroptosis through a multi-step mechanism. Firstly, Tim-AIII inhibits the growth of NSCLC cells, leading to a reduction in cell viability. Through computational predictions, HSP90 is identified as a target for Tim-AIII. The drug binds to HSP90, forming a complex that interacts with and degrades GPX4. Tim-AIII induces various hallmarks of ferroptosis, including increased lipid ROS, iron accumulation, MDA production, and GSH depletion. The HSP90-mediated degradation of GPX4 by Tim-AIII is confirmed through in vitro studies, where the drug-induced ferroptosis is blocked by an HSP90 inhibitor. In vivo, Tim-AIII demonstrates therapeutic potential by suppressing tumor growth in mouse models without significant toxicity [33]. Dihydroartemisinin (DHA), known for its potent GPX4 inhibitory properties, induces a notable shift in macrophage phenotype towards M1, particularly in the context of lung cancer. This shift is substantiated by the elevation of M1 markers, such as CD86, and an upsurge in the expression of pro-inflammatory cytokines like IL-1β, IL-6, and IL-12b. Macrophages treated with DHA also show increased levels of inducible nitric oxide synthase (iNOS) and guanylate-binding protein 5 (GBP5), indicating polarization towards the M1 phenotype. Additionally, these macrophages exhibit augmented capabilities in antigen presentation and phagocytosis, as indicated by heightened MHC-II expression and improved endocytic activity. Simultaneously, markers linked to the M2 phenotype are diminished. In vivo examinations in lung cancer tissues substantiate these outcomes, revealing macrophage infiltration and M1 reprogramming in lung tumor tissues upon DHA treatment [34]. Fuzheng Kang'ai Decoction (FZKA), a traditional Chinese herbal decoction comprising 12 components, exhibits inhibitory effects on NSCLC cell growth. The decoction suppresses the expression of GPX4 at both the protein and mRNA levels in NSCLC cells. This downregulation of GPX4 suggests a potential mechanism through which FZKA induces ferroptosis in NSCLC. Additionally, FZKA disrupts the system Xc^−^/glutathione axis, as seen by the downregulation of SLC7A11 and SLC3A2 components and a reduction in GSH levels [35]. Photodynamic Therapy (PDT) is a medical treatment that uses photosensitizing agents and light to selectively destroy targeted cells. In lung cancer, resistance to PDT can occur due to the up-regulation of GPX4, which degrades ROS essential for the therapy's effectiveness. DHA is proposed as a solution to overcome PDT resistance by inducing ferroptosis. By combining DHA with PDT, the inhibition of GPX4 allows for the accumulation of ROS during PDT, potentially enhancing its anti-cancer efficacy in lung cancer cells [36,37]. Many other products that are capable of targeting GPX4 activity are listed in Table 1 and depicted in Fig. 2.

### 2.3 FSP1

Ferroptosis suppressor protein 1 (FSP1) plays a crucial role in regulating ferroptosis. FSP1 functions independently of the traditional glutathione-dependent pathway, notably distinct from the lipid hydroperoxidase GPX4 system. Its primary role is to hinder ferroptosis through two principal pathways: the FSP1-coenzyme Q10 (CoQ10)-NAD(P)H axis and an atypical redox cycle involving vitamin K. In the former route, myristoylated FSP1 localizes to cellular membranes, utilizing NADH/NADPH to transform CoQ10 into its protective form, CoQ10H2, thereby preventing lipid peroxidation. In the latter, FSP1 functions as a vitamin K reductase, converting vitamin K to VKH2, an antioxidant. FSP1's engagement in thwarting ferroptosis extends to its role in the ESCRT–III–dependent membrane repair pathway. The protein undergoes intricate regulation influenced by various factors, including transcription factors, epigenetic modifications, and noncoding RNAs, thereby affecting its expression and influencing the development of diseases associated with ferroptosis. CoQ10 is primarily associated with mitochondrial function and is involved in the electron transport chain. The text emphasizes that FSP1's myristoylated form localizes to various cell membrane structures, such as the plasma membrane and mitochondria. By converting CoQ10 to its reduced form, CoQ10H2, FSP1 prevents lipid peroxidation, highlighting its role in protecting against ferroptosis [38]. A research explored the influence of curcumin on ferroptosis in A549 lung cancer cells, with a specific focus on CD133^+^ subgroups. CD133^+^ cells demonstrated higher tumorigenic potential than CD133^-^ cells. Treatment with curcumin led to a dose-dependent reduction in cell viability, downregulated the expression of GPX4 and FSP1 proteins, and induced morphological changes characteristic of ferroptosis. These effects were counteracted by Fer-1, an inhibitor of ferroptosis. In an in vivo study using NOD/SCID mice injected with A549 CD133^+^ cells, curcumin, RSL3, or iFSP1 treatments suppressed tumor growth, enhanced tumor pathology, and lowered Ki-67 expression. Curcumin displayed superior tumor-inhibiting effects compared to RSL3 or iFSP1, but Fer-1 administration reversed curcumin's anti-tumor effects. Examination of tumor tissues confirmed the downregulation of GPX4 and FSP1 by curcumin, and Fer-1 mitigated these effects. The results suggest that curcumin induces ferroptosis in CD133^+^ lung cancer cells, underscoring its potential as a therapeutic agent for targeting specific cancer subgroups [39].

### 2.4 Other transcription factors

Transcription factors exert a pivotal role in the regulation of ferroptosis. In addition to NRF2, several key transcription factors exist that influence ferroptosis [40].

#### 2.4.1 NRF2

Nuclear factor erythroid 2-related factor 2 (NRF2), a transcription factor known for its role in cellular defense against oxidative stress, plays a dual role in ferroptosis. Keap1 serves as a cytoplasmic sensor for various stresses, including oxidative stress, and functions as a negative regulator of NRF2. Under normal physiological conditions, NRF2 is bound to Keap1 in the cytoplasm. Keap1 acts as an adaptor for the E3 ubiquitin ligase complex, facilitating the ubiquitination and subsequent proteasomal degradation of NRF2. This constant turnover of NRF2 helps to maintain low intracellular levels of NRF2 and tightly regulate its activity. However, when cells encounter oxidative stress or other insults that disrupt redox balance, the interaction between NRF2 and Keap1 is altered. Reactive oxygen species (ROS) or electrophiles can modify cysteine residues within Keap1, leading to conformational changes that weaken its binding affinity for NRF2. As a result, NRF2 is released from Keap1 and stabilized, allowing it to translocate into the nucleus. Once in the nucleus, NRF2 forms heterodimers with small Maf proteins and binds to antioxidant response elements (AREs) in the promoter regions of target genes [41]. This binding activates the transcription of a battery of cytoprotective genes, including those encoding antioxidant enzymes, detoxification enzymes, and proteins involved in redox homeostasis. These target genes collectively promote the cellular defense against oxidative stress and maintain cellular viability under conditions of stress. In the context of lung cancer, dysregulation of the NRF2-Keap1 pathway has been implicated in tumor development and progression. Aberrant activation of NRF2 due to mutations in NRF2 or Keap1, or alterations in upstream signaling pathways, can lead to constitutive expression of antioxidant genes and enhanced resistance to oxidative stress within cancer cells. This heightened antioxidant capacity can contribute to tumor growth and survival by protecting cancer cells from the cytotoxic effects of reactive oxygen species and other stresses in the tumor microenvironment. Furthermore, emerging evidence suggests that the NRF2-Keap1 pathway may also play a role in regulating ferroptosis [42,43]. Under normal conditions, NRF2 is regulated by Keap1 and degraded, maintaining low intracellular levels. However, when cells face internal or external stress, such as oxidative stress, NRF2 is activated and dissociates from Keap1, translocating into the nucleus. Inside the nucleus, NRF2 orchestrates the transcription of antioxidant genes, which can counteract the initiation of ferroptosis by reducing ROS levels and preventing lipid peroxidation [44]. NRF2 plays a crucial role in the control of ferroptosis by impacting the expression of essential proteins, including GPX4 and SLC7A11. NRF2 indirectly adjusts GPX4 levels by coordinating the expression of genes involved in glutathione metabolism, supporting the necessary cofactors for GPX4's lipid repair function. Moreover, NRF2 directly governs SLC7A11, a crucial component of the cystine/glutamate transporter system Xc-, through binding to its promoter region. This regulatory mechanism ensures a sufficient cystine supply, a key precursor for glutathione synthesis, thereby bolstering cellular antioxidant defenses [45]. In addition to ferroptosis, NRF2 antioxidant response is involved in radiotherapy, immunotherapy, and drug resistances in lung cancer [[46], [47], [48], [49]]. Targeting NRF2 by traditional Chinese medicine has been reviewed previously [50]. Treatment with brusatol, a natural quassinoid compound derived from the Brucea javanica plant, primarily exerts its effects through the inhibition of the NRF2 pathway, a key regulator of cellular responses to oxidative stress. By suppressing NRF2, Brusatol reduces the cellular defense mechanisms against oxidative damage, leading to increased sensitivity to oxidative stress in lung cancer cells. This heightened vulnerability is particularly relevant in cancer cells, where elevated NRF2 activity is often associated with resistance to chemotherapy and rapid growth. Brusatol has demonstrated potential as an anticancer agent by sensitizing cells to oxidative damage, potentiating the effects of chemotherapy, and promoting ferroptosis. In vivo assays have also shown promising results, but further research is needed to fully understand Brusatol's clinical efficacy, safety, and potential side effects in human subjects [51]. Several evidence showed that natural NRF2 inhibitors can be used to improve the chemotherapy efficacy in lung cancer. Ni et al. investigated the therapeutic potential of combining manoalide (MA) with the EGFR-TKI osimertinib in lung cancer, particularly focusing on cells with KRAS mutations. MA exhibits inhibitory effects on cell proliferation, induces apoptosis, and prompts ferroptosis via oxidative stress and lipid peroxidation. Importantly, MA hinders the KRAS-ERK signaling pathway, activates the AMPK pathway, and disrupts the NRF2-SLC7A11 axis linked to ferroptosis resistance. The suggested combination of MA and osimertinib aims to synergistically heighten therapy sensitivity by concurrently targeting various cellular pathways, including MAPK signaling, and overcoming resistance mechanisms, such as those associated with KRAS mutations. This combined approach emerges as a promising strategy to tackle EGFR-TKI resistance and sensitize lung cancer cells, providing a comprehensive solution to navigate the intricacies of oncogenic signaling and cellular defense mechanisms [52]. Ginkgetin, derived from Ginkgo biloba leaves, amplifies the toxicity of cisplatin (DDP) in NSCLC cells. This co-treatment induces ferroptosis, characterized by heightened lipid peroxidation and intracellular free iron levels. Ginkgetin diminishes crucial ferroptosis regulators (SLC7A11 and GPX4 proteins), stimulates ROS generation, and suppresses the Nrf2/HO-1 antioxidant pathway activated by DDP. The inhibition of ferroptosis counteracts the cytotoxic effects, underscoring its significance. In a murine model, the ginkgetin + DDP combination substantially diminishes tumors, albeit partially reversed by a ferroptosis inhibitor. This investigation proposes that ginkgetin enhances DDP's anti-cancer impact in NSCLC by inducing ferroptosis and curtailing the Nrf2/HO-1 axis [53]. NRF2 inhibitors such as isoorientin [54], qingrehuoxue formula [55], ginkgetin [53], and brusatol [51] all have shown ferroptosis inducing (Fig. 1) capability and reduced tumor volume in vivo.

#### 2.4.2 STATs

Signal transducer and activator of transcription 3 (STAT3) is an oncogene that is aberrantly activated in about 70 % of cancers, promoting continuous transcription of growth factors and anti-apoptotic molecules for cell survival. Its hyperactivation involves various mechanisms, including receptor hyperactivation, elevated tyrosine kinase activity, loss of negative regulation, and cytokine stimulation. While somatic STAT3 mutations are less common, they occur in specific cancers. STAT3 regulates critical functions such as cell cycle progression, apoptosis inhibition, and modulation of immune responses, contributing to oncogenic transformation, cell motility, and anti-apoptotic factor expression. Targeting STAT3 is a therapeutic approach for cancer, inhibiting tumor growth and enhancing antitumor immunity [56]. Following activation by throsine kinase related pathways such as AKT, STAT3 expression is signficanlty increased, leading to SLC7A11 overexpression and ferroptosis resistance. Zerumbone is a naturally occurring compound derived from ginger rhizome, which can be used in combination with thyrosine kinase inhibitors like gefitinib for treatment of lung cancer, acting by inducing ferroptosis and inhibiting Akt/STAT3/SLC7A11 axis [57]. The combined treatment of Artemisia santolinifolia (AS) and Docetaxel (DTX) exhibits a synergistic impact on NSCLC by suppressing STAT3 signaling, resulting in reduced phosphorylation of STAT3 and consequent downregulation of survivin, a protein associated with cell survival. Particularly in A549 cells, the AS and DTX combination induces ferroptosis and increased ROS. This specific promotion of ferroptosis is evidenced by diminished cell viability, depletion of the antioxidant enzyme GPX4, and heightened ROS levels [58]. STAT1's function is similar to STAT3, affecting SLC7A11 expression in lung cancer. Epigallocatechin gallate (EGCG) is the main compound extracted from green tea with anti-cancer feature in NSCLC, acting as a potent inhibitor of proliferation, invasion and migration in cancer cells. In more detail, EGCG exerts these functions via suppressing the stimulatory effects of STAT1 on SLC7A11 promoter, leading to ferroptosis in A549 cells and restricting tumor growth in vivo [59].

#### 2.4.3 ATFs

Activating transcription factor (ATF) proteins, notably ATF3 and ATF4, play pivotal roles in regulating ferroptosis. ATF3 acts as a positive regulator, enhancing ferroptosis sensitivity by transcriptionally inhibiting key genes like SLC7A11. ATF4 has a dual role, influencing ferroptosis sensitivity in a cancer type-dependent manner. Depletion of ATF4 increases sensitivity in certain cancer cells, while its knockdown enhances ferroptosis induction in others [40]. In lung cancer, overactivation of ATF4 is positively linked with ferroptosis resistance, due to SLC7A11 regulation by this transcription factor. Fascaplysin is an extracted chemical from sponges that can induces ferroptosis in A549 cells by causing ER stress, leading to the activation of ATF4 [60]. Likewise, shikonin, a natural product isolated from Lithospermum erythrorhizon, exerts significant anti-small cell lung cancer (SCLC) effects by regulating ATF3 and inducing ferroptosis. Shikonin suppresses SCLC cell-derived xenograft growth in mice, correlating with increased ATF3 and decreased GPX4 expression. Mechanistically, shikonin upregulates ATF3 through the c-myc/HDAC1 axis, connecting its anti-SCLC effects to histone acetylation and ferroptosis induction [61].

## 3 Iron metabolism pathways as targets of natural products

**Fig. 3:**
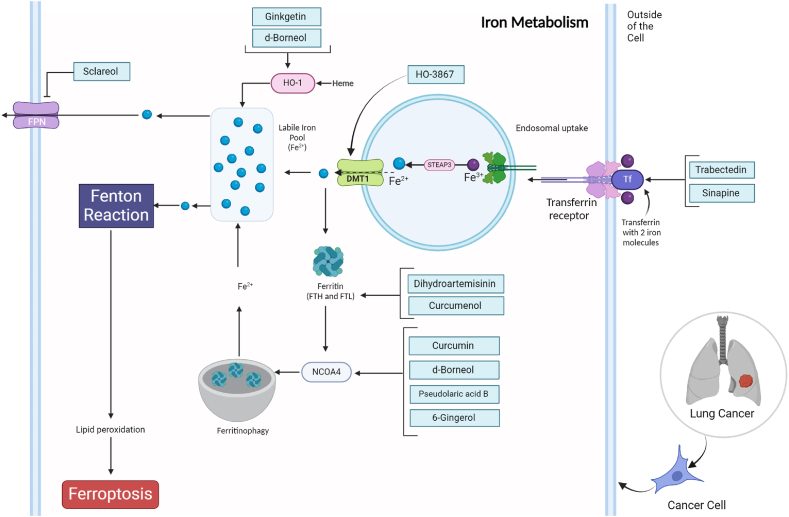
Deciphering the role of natural products in tumor suppression of lung cancer by affecting iron metabolism pathway and ferroptosis induction.

In ferroptosis iron metabolism plays a crucial role in driving cellular demise. Increased iron levels induce oxidative stress, initiating lipid peroxidation and eventual ferroptotic cell death. Proteins involved in iron metabolism, like transferrin, transferrin receptor, iron transporters, ferritin, and regulators like NCOA4, collectively impact ferroptosis sensitivity. Additionally, the interaction of autophagy, specifically ferritinophagy, with ferroptosis involves the modulation of iron availability. Targeting iron metabolism therapeutically, through natural products or manipulation of iron-related proteins, holds potential for regulating ferroptosis and alleviating its effects in diseases associated with this particular cell death pathway (Fig. 3) [83].

### 3.1 Transferrin receptor

Transferrin and its receptor play pivotal roles in ferroptosis by regulating iron transport into cells. Transferrin is a glycoprotein that binds to extracellular iron, and its receptor, transferrin receptor 1 (TFR1), facilitates the uptake of transferrin-bound iron into cells. In the context of ferroptosis, increased transferrin-bound iron uptake contributes to elevated intracellular iron levels. This excess iron promotes the Fenton reaction, leading to the production of ROS and subsequent lipid peroxidation, which are characteristic features of ferroptotic cell death. The interaction between transferrin, its receptor, and iron metabolism underscores the significance of these components in modulating the susceptibility of cells to ferroptosis [84]. Trabectedin, a marine-derived anti-cancer medication, induces ferroptosis in NSCLC cells by increasing the expression of TFR1. This process involves the activation of the HIF-1α/IRP1 axis by trabectedin, leading to the upregulation of TFR1. The drug's treatment enhances TFR1 expression, facilitating the uptake of iron through transferrin. The accumulated iron contributes to the iron-dependent cell death characteristic of ferroptosis. Silencing TFR1 diminishes the elevated iron levels induced by trabectedin and significantly reduces cell death in NSCLC, underscoring the crucial role of TFR1 in mediating ferroptosis triggered by trabectedin [70]. While Trabectedin exerts anti-cancer effects and ferroptosis regardless of p53 activity status, some alkaloid natural products such as Sinapine require intact p53 for inducing ferroptosis. Sinapine treatment leads to a dose-dependent increase in TF, TFR1, and the tumor suppressor protein p53. TF and TFR1 are integral components of cellular iron uptake, suggesting a potential involvement of iron metabolism in Sinapine-induced ferroptosis. Knockdown experiments targeting TF and TFR1 demonstrate their crucial roles in modulating cell viability, cell death, and the associated changes in iron levels, lipid peroxidation, and ROS [71,85].

### 3.2 DMT1

Divalent metal transporter 1 (DMT1), known as SLC7A2, is a crucial protein engaged in assimilating dietary iron inside cells. Once absorbed, ferrous iron can bind to transferrin inside the enterocyte, forming a complex that is internalized via endocytosis. Inside endosomes, the iron is released from transferrin, and DMT1 plays a role in transporting this iron from the endosome into the cytoplasm of the cell. DMT1 is present in diverse cell types, playing a key role in maintaining cellular iron balance. In the realm of ferroptosis, DMT1's participation in iron absorption and potential engagement in intracellular transport mechanisms could contribute to the initiation of pathways leading to ferroptosis [86,87]. HO-3867, a novel diarylidenyl piperidone analog, induces ferroptosis in NSCLC cells through a multifaceted mechanism. Treatment with HO-3867 leads to the upregulation of DMT1 in a p53-dependent manner, resulting in the accumulation of iron within NSCLC cells. This iron accumulation is pivotal for the induction of ferroptosis, characterized by iron-dependent lipid peroxidation. Concurrently, HO-3867 promotes a dosage-dependent generation of ROS, and this ROS production is found to be iron-dependent. DMT1 inhibitors and DMT1 silencing attenuate both iron accumulation and cell death induced by HO-3867. Additionally, HO-3867 downregulates GPX4, leading to a reduction in GSH levels. Overexpression of GPX4 counteracts the effects of HO-3867, protecting against cell death. In summary, HO-3867 orchestrates ferroptosis in NSCLC cells by modulating DMT1, iron homeostasis, ROS generation, and GPX4 levels [72].

### 3.3 Ferroportin

Ferroportin (FPN) is a critical protein involved in cellular iron transport, acting as the primary exporter of iron from cells to maintain iron homeostasis. In the context of ferroptosis, FPN role lies in its ability to regulate intracellular iron levels. By facilitating the efflux of iron from cells, FPN can influence the susceptibility of cells to ferroptotic stimuli. The intricate regulation of FPN expression and activity makes it a key player in cellular responses to iron-induced stress and a potential target for therapeutic interventions in conditions associated with disrupted iron homeostasis and ferroptosis [86,88]. Sclareol (SC), a labdane diterpenoid extracted from Salvia sclarea, leads to changes in proteins associated with iron metabolism, notably a reduction in FPN expression in NSCLC. FPN plays a crucial role in exporting cellular iron, and the decrease in its expression might be a factor in the observed buildup of iron and the initiation of ferroptosis in A549 cells following SC treatment [73].

### 3.4 FTH

Ferritin, a central participant in iron regulation within cells, plays a pivotal role in the initiation of ferroptosis by modulating cellular iron concentrations. Functioning as an iron reservoir, ferritin prevents excess iron from engaging in iron-dependent reactions that result in oxidative harm. Comprising heavy (FTH) and light (FTL) chain subunits, ferritin constructs a protective structure that stores surplus iron intracellularly. The reduction in FTH, specifically the heavy chain component, can elevate labile iron levels, instigating the Fenton reaction and subsequent oxidative stress associated with ferroptosis. This expanded pool of free iron facilitates the Fenton reaction, generating ROS that contribute to lipid peroxidation, ultimately culminating in cell death through ferroptosis. Consequently, the modulation of ferritin expression stands as a critical determinant in upholding iron balance and shaping cellular susceptibility to ferroptotic pathways [83,89]. After exposure to dihydroartemisinin in NSCLC, the levels of ferroptosis-associated proteins FTH was notably reduced, resulting in the release of unbound iron [63]. Curcumenol, an organic compound derived from Curcuma wenyujin, exhibits a notable ability to combat lung cancer by selectively inducing ferroptotic cell death. It displays preferential toxicity toward lung cancer cells, restraining their viability and growth while sparing normal lung cells. The primary mode of cell death induced by curcumenol is ferroptosis, marked by alterations in crucial ferroptosis-associated factors, including a reduction in FTH1 expression, leading to the release of free iron. This treatment results in changes in oxidative stress markers, intracellular ROS, and GSH, all indicative of ferroptosis initiation. In an in vivo lung cancer xenograft model, curcumenol hinders tumor growth through ferroptosis induction, a process reversed by the ferroptosis inhibitor deferoxamine (DFO). Furthermore, the involvement of the lncRNA H19/miR-19b-3p/FTH1 axis in curcumenol's ferroptotic effects suggests a specific molecular mechanism underlying its impact on lung cancer cells [74].

### 3.5 NCOA4

Selective autophagy mediated by nuclear receptor coactivator 4 (NCOA4), known as ferritinophagy, is a specialized cellular process that specifically breaks down ferritin, the primary storage form of iron in cells. NCOA4, a protein, plays a vital role in this process by interacting with ferritin. When the cell requires iron, the NCOA4-ferritin complex is directed to autophagosomes, cellular structures that encapsulate materials slated for degradation. These autophagosomes then merge with lysosomes, where ferritin undergoes degradation, releasing iron into the cell. This precisely controlled mechanism helps maintain cellular iron levels, preventing both iron deficiency and excess. Dysregulation of NCOA4-mediated ferritinophagy is associated with diseases related to iron metabolism and oxidative stress, including cancers, leading to ferroptosis [90]. Several NCOA4 modulators, including 6-Gingerol, Pseudolaric acid B, d-Borneol, and Curcumin, have been found that can increase its expression and leads to ferritinophagy and ferroptosis induction (Table 1). The collaboration of d-borneol and cisplatin exhibits a synergistic effect in overcoming cisplatin resistance in NSCLC by instigating ferroptosis through diverse mechanisms. In a xenograft tumor model, the combined treatment substantially diminishes tumor growth and weight, demonstrating superior antitumor efficacy compared to individual treatments. RNA sequencing reveals the ferroptosis pathway as a pivotal contributor to this synergistic effect. The combination induces oxidative damage, heightens ROS, and facilitates lipid peroxidation, signifying ferroptotic cell death. The adjustment of ferroptosis-associated genes, including ACSL4, PCBP2, PRNP, and NCOA4, is observed, underscoring NCOA4's crucial role in ferritinophagy. Additionally, the combination stimulates autophagy and restrains the epithelial-mesenchymal transition (EMT), collectively enhancing sensitivity to cisplatin. These findings elucidate a comprehensive mechanism involving ferroptosis, autophagy, and EMT modulation to surmount cisplatin resistance in NSCLC cells [77].

## 4 Lipid pathways as a target of natural products

Lipid metabolism intricately regulates ferroptosis, a form of programmed cell death characterized by lipid peroxidation. The cellular membrane composition, particularly the presence of polyunsaturated fatty acids (PUFAs), significantly influences the susceptibility to ferroptosis. The process involves the peroxidation of lipids due to the interaction of PUFAs with reactive oxygen species and ferrous iron, resulting in membrane disruption and cell demise. Various metabolic enzymes involved in PUFA synthesis, elongation, and desaturation, as well as those influencing monounsaturated fatty acids (MUFAs), contribute to the sensitivity of cells to ferroptosis. Ether lipids, phospholipid synthesis, and remodeling processes also play essential roles in determining the cellular response to ferroptosis. Furthermore, lipid uptake and storage mechanisms, such as fatty acid transporters and lipid droplets, influence the occurrence of ferroptosis. In therapeutic contexts, manipulating lipid metabolism is seen as a strategy, particularly in the treatment of cancer (Fig. 1). A comprehensive understanding of these lipid metabolism intricacies is vital for unraveling ferroptosis mechanisms and developing targeted interventions for various diseases [91].

### 4.1 ACSL4

ACSL4, or Acyl-CoA synthetase long-chain family member 4, is an enzyme crucial in ferroptosis. ACSL4 facilitates the esterification of PUFAs to CoA, generating PUFA-CoA esters that are incorporated into glycerol-3-phosphate, forming PUFA-containing phospholipids. These phospholipids, particularly those with arachidonic acid or adrenic acid, are highly susceptible to peroxidation by redox-active iron, leading to lipid peroxide accumulation and eventual cell death through ferroptosis. While ACSL4 has been traditionally considered a universal regulator of ferroptosis, recent research indicates that its role is context-dependent, being more crucial in ferroptosis induced by GPX4 inhibitors compared to other ferroptosis-inducing conditions like SLC7A11 inhibition or cystine starvation. This contextual variability underscores the intricate regulation of ferroptosis in diverse cellular environments [92]. Scoparone, a natural compound extracted from Artemisia capillaris Thunb, exhibits selective anti-tumor effects against NSCLC cells by diminishing cell viabilities, hindering invasion and migration, and instigating apoptosis and ferroptosis. In the apoptotic pathway, scoparone reduces Mcl-1 levels via FBW7-mediated ubiquitination, activates Bax, and induces cell death. In the ferroptotic pathway, scoparone amplifies lipid peroxidation, iron, and ROS levels, elevating ACSL4 through the SP1/ACSL4 axis. ACSL4 depletion mitigates scoparone-induced cell death and lipid peroxidation, underscoring its involvement in ferroptosis. The JNK/SP1 pathway is triggered by scoparone, with JNK inhibitors alleviating cell death and suppressing ACSL4 and SP1 elevation. Furthermore, scoparone-induced ROS production contributes to Bax activation and the JNK/SP1/ACSL4 axis. This detailed mechanism underscores scoparone's dual promotion of apoptosis and ferroptosis in NSCLC cells through intricate molecular pathways, emphasizing its potential in cancer therapy [79,93].

## 5 Mitochondria as a target of natural products

Mitochondria play a pivotal role in the process of ferroptosis. Throughout ferroptosis, mitochondria undergo morphological alterations characterized by shrinkage and modified dynamics. The integrity of both outer and inner mitochondrial membranes is compromised, facilitating the occurrence of lipid peroxidation specifically on phospholipids containing polyunsaturated fatty acids. Mitochondria serve as prominent sources of ROS, thereby contributing to heightened oxidative stress. Additionally, these organelles are central to cellular iron metabolism, where an excess of mitochondrial iron can instigate ROS generation through the Fenton reaction. The regulation of ferroptosis is further influenced by mitochondrial calcium dynamics, amino acid metabolism (such as glutaminolysis), and the participation of NEET proteins. The intricate interplay among these mitochondrial functions underscores their critical role in initiating and advancing the process of ferroptosis [94].

### 5.1 VDACs

**Fig. 4:**
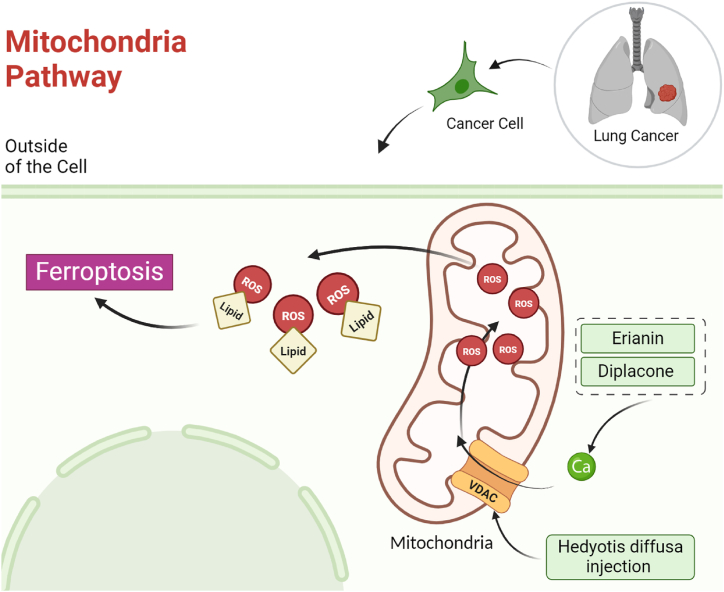
Deciphering the role of natural products in tumor suppression of lung cancer by affecting mitochondria pathway and ferroptosis induction.

Voltage-dependent anion channels (VDACs) regulate ferroptosis by influencing mitochondrial function and redox homeostasis. As integral components of the outer mitochondrial membrane, VDACs facilitate the exchange of ions and metabolites between the cytoplasm and mitochondria. In the context of ferroptosis, VDACs regulate mitochondrial membrane permeability, crucial for maintaining mitochondrial integrity during the process. This regulation impacts the flux of ions and metabolites, influencing the cellular redox status. Furthermore, VDACs may interact with the permeability transition pore (PTP), a mitochondrial pore associated with cell death, potentially modulating the susceptibility of cells to ferroptosis. Overall, VDACs contribute significantly to ferroptosis by modulating mitochondrial permeability, redox balance, and potentially influencing the permeability transition pore [95]. In lung cancer, VDAC gene expression is negatively correlated with survival of patients [96]. Hedyotis diffusa injection (HDI), a traditional Chinese herbal medicine extracted from Hedyotis diffusa, exhibits strong anti-tumor properties in lung adenocarcinoma by triggering ferroptosis. HDI hinders cancer cell proliferation, colony formation, invasion, and migration. The induction of ferroptosis is evidenced by changes in cell structure and elevated levels of ferroptosis-related markers, including lipid ROS, Fe2+, and MDA. Although HDI doesn't directly involve the GPX4 and PUFA-PLS pathways, it significantly influences the VDAC2/3 pathway by regulating the expression of Bcl2 and Bax. In vivo experiments in a xenograft tumor model confirm that HDI effectively restrains tumor growth with fewer adverse effects compared to cisplatin, highlighting increased ferroptosis levels in tumor tissues. Overall, HDI emerges as a promising therapeutic agent for lung adenocarcinoma, exerting its effects by inducing ferroptotic cell death through modulation of VDAC2/3 activity via Bcl2 and Bax adjustments (Fig. 4) [80].

### 5.2 Mitochondrial Ca^2+^ influx

Mitochondrial calcium overload is a crucial contributor to ferroptosis. The influx of calcium into mitochondria induces the generation of ROS, initiating lipid peroxidation. Additionally, mitochondrial calcium overload leads to the opening of the mitochondrial permeability transition pore (MPTP), disrupting mitochondrial function and membrane potential. This disruption facilitates the release of pro-apoptotic factors, including cytochrome *c*, into the cytoplasm, further promoting the execution of ferroptosis. The interplay between mitochondrial calcium and iron metabolism enhances susceptibility to ferroptosis, as iron is intimately involved in lipid peroxidation. Overall, mitochondrial calcium overload plays a pivotal role in creating a pro-ferroptotic environment, contributing to the cascade of events leading to ferroptotic cell death [97]. Researchers explored the impact of diplacone (DP) isolated from Paulownia tomentosa fruit on A549 human non-small lung carcinoma cells. DP treatment led to a dose-dependent inhibition of cell growth, and notably, induced a unique form of nonapoptotic cell death characterized by necrosis rather than apoptosis. DP triggered an increase in cytosolic and mitochondrial Ca^2+^ levels, resulting in mitochondrial dysfunction, ROS generation, loss of mitochondrial membrane potential, and mitochondrial permeability transition (MPT) pore-opening. Intriguingly, DP-induced cell death exhibited characteristics of ferroptosis, evidenced by increased lipid peroxidation and upregulation of ATF3, a key gene in ferroptosis. Inhibition of ferroptosis alleviated DP-induced cell death and associated mitochondrial changes. Furthermore, DP treatment led to the release of high-mobility group box 1 protein (HMGB1), a marker of immunogenic cell death, enhancing the cytotoxicity of natural killer (NK) cells. This comprehensive study sheds light on the intricate cellular mechanisms through which DP induces ferroptosis in A549 cells, linking Ca^2+^ dysregulation, mitochondrial dysfunction, and immunogenic cell death [82]. Erianin, a potent agent against lung cancer cells, manifests diverse effects, including the initiation of cell death, hindrance of proliferation, and deterrence of migration. Its application results in heightened suppression of lung cancer cell growth and imposition of G2/M-phase cell cycle arrest. Remarkably, erianin induces ferroptosis, characterized by increased intracellular ROS, GSH depletion, and lipid peroxidation, accompanied by reduced chelatable iron levels. Furthermore, the study suggests the involvement of calcium/calmodulin signaling in erianin-induced ferroptosis, evident in elevated intracellular Ca^2+^ concentrations and calmodulin activation post-erianin treatment. This implies a potential modulation of Ca^2+^/CaM signaling by erianin, contributing to its ferroptotic effects. In vivo experiments underscore the therapeutic promise of erianin, showcasing its ability to curb tumor growth and impede metastasis, highlighting its potential as a comprehensive anti-cancer agent against lung cancer cells [81]. Similarly, the previously discussed manoalide (MA) that can induce ferroptosis of lung cancer through NRF2-SLC7A11 and FTH1 pathways, can also affect mitochondrial Ca^2+^ overload (Fig. 4) [52].

## 6 Non-coding RNAs, and lung cancer ferroptosis

In the quest for novel therapeutic approaches, the interaction between natural compounds, ferroptosis, and non-coding RNAs (ncRNAs) has garnered significant attention. Natural compounds such as curcumenol and cinobufotalin have shown potential in inducing ferroptosis in lung cancer cells, offering promising avenues for treatment. Curcumenol, an active ingredient in Wenyujin, has demonstrated significant antitumor effects by inducing ferroptosis in lung cancer cells. Research has shown that curcumenol effectively induces cell death and suppresses cell proliferation both in vitro and in vivo. RNA sequencing revealed that treatment with curcumenol significantly downregulated the long non-coding RNA H19 (lncRNA H19) in lung cancer cells. The overexpression of lncRNA H19 countered the anticancer effects of curcumenol, while knockdown of lncRNA H19 enhanced ferroptosis induced by curcumenol. Mechanistically, lncRNA H19 functions as a competing endogenous RNA (ceRNA) for miR-19b-3p, which regulates the transcription of ferritin heavy chain 1 (FTH1), a key marker of ferroptosis. This lncRNA H19/miR-19b-3p/FTH1 axis is crucial for mediating curcumenol-induced ferroptotic cell death, highlighting the role of ncRNAs in modulating the effects of natural compounds on ferroptosis in lung cancer [74,98].

Similarly, cinobufotalin, a traditional Chinese medicine isolated from dried toad venom, has shown efficacy in treating lung cancer through its active component, resibufogenin. Cinobufotalin was found to suppress lung cancer cell growth and induce ferroptosis. Mechanistic studies indicated that cinobufotalin increases the level of long non-coding RNA LINC00597 and downregulates hsa-miR-367-3p expression in lung cancer cells via resibufogenin. Further analysis revealed that the ferroptosis inducer transferrin receptor (TFRC) is a target of hsa-miR-367-3p. LINC00597 upregulates TFRC expression by sponging hsa-miR-367-3p, thereby promoting ferroptosis in lung cancer cells. This LINC00597/hsa-miR-367-3p/TFRC pathway elucidates the intricate regulatory network involving ncRNAs in cinobufotalin-induced ferroptosis [99].

These findings underscore the significant role of ncRNAs in the interplay between natural compounds and ferroptosis in lung cancer cells. While natural compounds like curcumenol and cinobufotalin exhibit potential in inducing ferroptosis, their interactions with ncRNAs are crucial in modulating these effects. Comprehensive mechanistic studies and clinical research are essential to fully understand these interactions and translate these preclinical findings into effective therapeutic strategies for lung cancer treatment. The exploration of natural compounds in combination with ncRNA modulation could pave the way for innovative and more effective treatments, addressing the urgent need for novel drugs and approaches in lung cancer therapy.

## 7 Endoplasmic stress and lung cancer feroptosis

Endoplasmic reticulum (ER) stress, on the other hand, is a cellular condition where unfolded or misfolded proteins accumulate within the ER, leading to cellular dysfunction and often cell death. In the context of lung cancer, where both ferroptosis and ER stress are implicated, targeting ER stress as a means to induce ferroptosis has emerged as a promising strategy. Natural products, compounds derived from plants, fungi, or marine organisms, have shown potential in modulating both ferroptosis and ER stress pathways. Several natural products have been identified as potential inducers of ferroptosis in lung cancer cells by targeting ER stress pathways [100,101].

Manoalide (MA), a natural inhibitor of phospholipase A2 (PLA2), has garnered attention for its anticancer properties. Recent studies have delved into its mechanism of action, particularly in the context of EGFR-TKI-resistant lung cancer. By utilizing KRAS-mutated lung cancer cells and organoids, researchers demonstrated MA's ability to inhibit proliferation and induce endoplasmic reticulum (ER) stress via a ROS-dependent mechanism. ER stress, a cellular response to disturbances in protein folding within the ER, plays a pivotal role in MA-induced cytotoxicity. The accumulation of misfolded proteins triggers the unfolded protein response (UPR), a signaling pathway aimed at restoring ER homeostasis or initiating apoptosis if the stress is severe and prolonged. In the context of lung cancer, MA-induced ER stress not only sensitizes KRAS-mutated cells to osimertinib but also disrupts mitochondrial function, leading to lipid peroxidation and ultimately ferroptosis. This multifaceted effect underscores the importance of ER stress modulation in enhancing therapeutic efficacy and overcoming drug resistance in lung cancer treatment [52,102].

Melittin, a natural polypeptide found in bee venom, has exhibited significant antitumor activity, particularly in lung cancer. Studies have elucidated its mechanism of action in A549 lung carcinoma cells, revealing its induction of reactive oxygen species (ROS) and intracellular iron accumulation. These events disrupt the glutathione-glutathione peroxidase 4 antioxidant system, leading to lipid peroxidation and ultimately triggering ferroptosis. Furthermore, melittin activates ER stress-mediated apoptotic signaling, highlighting its multifaceted approach to inducing cell death in lung cancer cells. Ferroptosis inhibitors such as ferrostatin-1 and deferoxamine have been shown to reverse melittin-induced cell death, further underscoring the involvement of ferroptosis in its antitumor effects [65].

Docosahexaenoic acid (DHA), a bioactive omega-3 fatty acid, has demonstrated remarkable potential in promoting immunogenic cell death (ICD) in lung cancer. Through a series of experiments involving Lewis lung cancer cells (LLC) and A549 cells, researchers unveiled DHA's ability to enhance apoptosis and immunogenicity while inducing lipid peroxide accumulation and ferroptosis. Notably, DHA-induced ER stress and DNA damage were found to be integral to its immunogenic effects, with ferroptosis inhibitors attenuating these processes and diminishing immunogenicity. Conversely, exogenous iron ions further augmented DHA-induced immunogenicity, emphasizing the interplay between ferroptosis, ER stress, and the immune response. In preclinical models, DHA exhibited potent antitumor efficacy, highlighting its potential as a promising therapeutic agent for lung cancer [103].

## 8 Conclusion

The induction of ferroptosis by natural products in lung cancer holds promise as an innovative therapeutic strategy. Lung cancer, a global health challenge with limited treatment success, necessitates novel approaches for improved outcomes. Natural products, including compounds from TCM, offer diverse mechanisms such as apoptosis induction, cell cycle arrest, and anti-inflammatory effects. Cancer cells, particularly in lung cancer, exhibit heightened vulnerability to ferroptosis due to their increased dependence on iron. Natural products may exploit this susceptibility, selectively eliminating cancer cells while minimizing harm to normal cells and potentially overcoming resistance to conventional treatments. Natural products exert their influence on diverse pathways in lung cancer, inducing ferroptosis through modulation of antioxidant pathways (System Xc^−^, GPX4, NRF2, FSP1), iron metabolism regulators (TFRC, DMT1, HO-1, Ferritin, NCOA4), lipid metabolism regulators (ACSL4), and mitochondria-dependent processes (VDAC, Calcium influx). These compounds disrupt the cystine/glutamate antiporter (System Xc^−^) to limit glutathione synthesis, inhibit GPX4, modulate NRF2 activity, and impact FSP1 function. They also regulate iron uptake (TFRC), transport (DMT1), heme degradation (HO-1), ferritin storage, and ferritinophagy (NCOA4). In lipid metabolism, natural products target ACSL4, inducing lipid peroxidation. Mitochondrial influence involves modulation of VDAC and calcium influx, collectively offering a comprehensive strategy for ferroptosis induction in lung cancer. Further exploration of these abbreviated gene targets and their interactions is crucial for advancing our understanding of the therapeutic potential of natural products in lung cancer treatment.

## Funding

No fund was received for this study.

## Availability of data and material

The original contributions presented in the study are included in the article. Further inquiries can be directed to the corresponding author.

## Conflicts of interest/competing interests

The authors have no conflicts of interest to declare.

## Informed consent to consent for publication

Not applicable (Review Study).

## Ethics approval and consent to participate

Not applicable (Review Study).

## CRediT authorship contribution statement

**Wang Yuhao:** Writing – review & editing, Writing – original draft. **Cheng Shenghua:** Writing – review & editing, Writing – original draft. **Chen Jueying:** Writing – review & editing, Writing – original draft. **Xiang Shate:** Writing – review & editing, Writing – original draft. **Song Rongrong:** Writing – review & editing, Writing – original draft. **Shen Xiangfeng:** Writing – review & editing, Writing – original draft.

## Declaration of generative AI and AI-assisted technologies in the writing process

During the preparation of this work, the authors used ChatGPT by OpenAI to improve paper readability. After using this tool/service, the authors reviewed and edited the content as needed and took full responsibility for the publication's content.

## Declaration of competing interest

The authors declare that they have no known competing financial interests or personal relationships that could have appeared to influence the work reported in this paper.

## References

[1] Sung H., Ferlay J., Siegel R.L., Laversanne M., Soerjomataram I., Jemal A. (2021). Global cancer statistics 2020: GLOBOCAN estimates of incidence and mortality worldwide for 36 cancers in 185 countries. CA A Cancer J. Clin..

[2] Panakkal N., Lekshmi A., Saraswathy V.V., Sujathan K. (2023). Effective lung cancer control: an unaccomplished challenge in cancer research. CytoJournal.

[3] Vicidomini G. (2023). Current challenges and future advances in lung cancer: genetics, instrumental diagnosis and treatment. Cancers.

[4] Ebrahimnezhad M., Natami M., Bakhtiari G.H., Tabnak P., Ebrahimnezhad N., Yousefi B. (2023). FOXO1, a tiny protein with intricate interactions: promising therapeutic candidate in lung cancer. Biomed. Pharmacother..

[5] Wattanathamsan O., Hayakawa Y., Pongrakhananon V. (2019). Molecular mechanisms of natural compounds in cell death induction and sensitization to chemotherapeutic drugs in lung cancer. Phyther Res.

[6] Gao T.-H., Liao W., Lin L.-T., Zhu Z.-P., Lu M.-G., Fu C.-M. (2022). Curcumae rhizoma and its major constituents against hepatobiliary disease: pharmacotherapeutic properties and potential clinical applications. Phytomedicine.

[7] Su X.-L., Wang J.-W., Che H., Wang C.-F., Jiang H., Lei X. (2020). Clinical application and mechanism of traditional Chinese medicine in treatment of lung cancer. Chin Med J (Engl).

[8] Li Z., Feiyue Z., Gaofeng L. (2021). Traditional Chinese medicine and lung cancer——from theory to practice. Biomed. Pharmacother..

[9] Xiang J., Mlambo R., Shaw I., Seid Y., Shah H., He Y. (2023). Cryopreservation of bioflavonoid-rich plant sources and bioflavonoid-microcapsules: emerging technologies for preserving bioactivity and enhancing nutraceutical applications. Front. Nutr..

[10] Huang B., Gui M., An H., Shen J., Ye F., Ni Z. (2023). Babao Dan alleviates gut immune and microbiota disorders while impacting the TLR4/MyD88/NF-кB pathway to attenuate 5-Fluorouracil-induced intestinal injury. Biomed. Pharmacother..

[11] Wang X., Feng Y., Wang N., Cheung F., Tan H.Y., Zhong S. (2014). Chinese medicines induce cell death: the molecular and cellular mechanisms for cancer therapy. BioMed Res. Int..

[12] Meng Z., Tan Y., Duan Y., Li M. (2024). Monaspin B, a novel cyclohexyl-furan from cocultivation of Monascus purpureus and Aspergillus oryzae, exhibits potent antileukemic activity. J. Agric. Food Chem..

[13] Dixon S.J., Lemberg K.M., Lamprecht M.R., Skouta R., Zaitsev E.M., Gleason C.E. (2012). Ferroptosis: an iron-dependent form of nonapoptotic cell death. Cell.

[14] Zhang C., Liu X., Jin S., Chen Y., Guo R. (2022). Ferroptosis in cancer therapy: a novel approach to reversing drug resistance. Mol. Cancer.

[15] Xing N., Du Q., Guo S., Xiang G., Zhang Y., Meng X. (2023). Ferroptosis in lung cancer: a novel pathway regulating cell death and a promising target for drug therapy. Cell Death Dis..

[16] Tabnak P., HajiEsmailPoor Z., Soraneh S. (2021). Ferroptosis in lung cancer: from molecular mechanisms to prognostic and therapeutic opportunities. Front. Oncol..

[17] Rosell R., Jain A., Codony-Servat J., Jantus-Lewintre E., Morrison B., Ginesta J.B. (2023). Biological insights in non-small cell lung cancer. Cancer Biol Med.

[18] Wei X., Li X., Hu S., Cheng J., Cai R. (2023). Regulation of ferroptosis in lung adenocarcinoma. Int. J. Mol. Sci..

[19] Tang D., Chen X., Kang R., Kroemer G. (2021). Ferroptosis: molecular mechanisms and health implications. Cell Res..

[20] Li F.-J., Long H.-Z., Zhou Z.-W., Luo H.-Y., Xu S.-G., Gao L.-C. (2022). System Xc−/GSH/GPX4 axis: an important antioxidant system for the ferroptosis in drug-resistant solid tumor therapy. Front. Pharmacol..

[21] Wang X., Chen Y., Wang X., Tian H., Wang Y., Jin J. (2021). Stem cell factor SOX2 confers ferroptosis resistance in lung cancer via upregulation of SLC7A11. Cancer Res..

[22] Zhang W., Sun Y., Bai L., Zhi L., Yang Y., Zhao Q. (2021). RBMS1 regulates lung cancer ferroptosis through translational control of SLC7A11. J. Clin. Invest..

[23] Peng Y., Ouyang L., Zhou Y., Lai W., Chen Y., Wang Z. (2023). AhR promotes the development of non-small cell lung cancer by inducing SLC7A11-dependent antioxidant function. J. Cancer.

[24] Du J., Krishnamoorthy K., Ramabhai V., Yang D. (2023). Targeting ferroptosis as a therapeutic implication in lung cancer treatment by a novel naphthoquinone inducer: juglone. Mol. Biotechnol..

[25] Chen P., Lv X., Zheng Z. (2024). Gigantol exerts anti-lung cancer activity by inducing ferroptosis via SLC7A11-GPX4 axis. Biochem. Biophys. Res. Commun..

[26] Jiaqi L., Siqing H. (2023). Andrographolide promoted ferroptosis to repress the development of non-small cell lung cancer through activation of the mitochondrial dysfunction. Phytomedicine.

[27] Xu W., Yu H. (2022). Valtrate antagonizes malignant phenotypes of lung cancer cells by reducing SLC7A11. Hum. Exp. Toxicol..

[28] Liu X.-Y., Wei D.-G., Li R.-S. (2022). Capsaicin induces ferroptosis of NSCLC by regulating SLC7A11/GPX4 signaling in vitro. Sci. Rep..

[29] Zeng Y.-Y., Luo Y.-B., Ju X.-D., Zhang B., Cui Y.-J., Pan Y.-B. (2022). Solasonine causes redox imbalance and mitochondrial oxidative stress of ferroptosis in lung adenocarcinoma. Front. Oncol..

[30] Xu C., Jiang Z.-B., Shao L., Zhao Z.-M., Fan X.-X., Sui X. (2023). β-Elemene enhances erlotinib sensitivity through induction of ferroptosis by upregulating lncRNA H19 in EGFR-mutant non-small cell lung cancer. Pharmacol. Res..

[31] Seibt T.M., Proneth B., Conrad M. (2019). Role of GPX4 in ferroptosis and its pharmacological implication. Free Radic. Biol. Med..

[32] Zhao L.-P., Wang H.-J., Hu D., Hu J.-H., Guan Z.-R., Yu L.-H. (2023). β-Elemene induced ferroptosis via TFEB-mediated GPX4 degradation in EGFR wide-type non-small cell lung cancer. J. Adv. Res..

[33] Zhou C., Yu T., Zhu R., Lu J., Ouyang X., Zhang Z. (2023). Timosaponin AIII promotes non-small-cell lung cancer ferroptosis through targeting and facilitating HSP90 mediated GPX4 ubiquitination and degradation. Int. J. Biol. Sci..

[34] Li L.-G., Peng X.-C., Yu T.-T., Xu H.-Z., Han N., Yang X.-X. (2022). Dihydroartemisinin remodels macrophage into an M1 phenotype via ferroptosis-mediated DNA damage. Front. Pharmacol..

[35] Zhao Y.-Y., Yang Y.-Q., Sheng H.-H., Tang Q., Han L., Wang S.-M. (2022). GPX4 plays a crucial role in Fuzheng Kang’ai decoction-induced non-small cell lung cancer cell ferroptosis. Front. Pharmacol..

[36] Han N., Li L.-G., Peng X.-C., Ma Q.-L., Yang Z.-Y., Wang X.-Y. (2022). Ferroptosis triggered by dihydroartemisinin facilitates chlorin e6 induced photodynamic therapy against lung cancer through inhibiting GPX4 and enhancing ROS. Eur. J. Pharmacol..

[37] Liu K., Jiang Z., Lalancette R.A., Tang X., Jäkle F. (2022). Near-infrared-absorbing B-N Lewis pair-functionalized anthracenes: electronic structure tuning, conformational isomerism, and applications in photothermal cancer therapy. J. Am. Chem. Soc..

[38] Li W., Liang L., Liu S., Yi H., Zhou Y. (2023). FSP1: a key regulator of ferroptosis. Trends Mol. Med..

[39] Zhou J., Zhang L., Yan J., Hou A., Sui W., Sun M. (2023). Curcumin induces ferroptosis in A549 CD133+ cells through the GSH-GPX4 and FSP1-CoQ10-NAPH pathways. Discov. Med..

[40] Dai C., Chen X., Li J., Comish P., Kang R., Tang D. (2020). Transcription factors in ferroptotic cell death. Cancer Gene Ther..

[41] Suzuki T., Takahashi J., Yamamoto M. (2023). Molecular basis of the KEAP1-NRF2 signaling pathway. Mol. Cell..

[42] Adinolfi S., Patinen T., Jawahar Deen A., Pitkänen S., Härkönen J., Kansanen E. (2023). The KEAP1-NRF2 pathway: targets for therapy and role in cancer. Redox Biol..

[43] Ulasov A.V., Rosenkranz A.A., Georgiev G.P., Sobolev A.S. (2022). Nrf2/Keap1/ARE signaling: towards specific regulation. Life Sci..

[44] Yan R., Lin B., Jin W., Tang L., Hu S., Cai R. (2023). NRF2, a superstar of ferroptosis. Antioxidants.

[45] Song X., Long D. (2020). Nrf2 and ferroptosis: a new research direction for neurodegenerative diseases. Front. Neurosci..

[46] Sun X., Dong M., Li J., Sun Y., Gao Y., Wang Y. (2024). NRF2 promotes radiation resistance by cooperating with TOPBP1 to activate the ATR-CHK1 signaling pathway. Theranostics.

[47] Zavitsanou A.-M., Pillai R., Hao Y., Wu W.L., Bartnicki E., Karakousi T. (2023). KEAP1 mutation in lung adenocarcinoma promotes immune evasion and immunotherapy resistance. Cell Rep..

[48] Qian S., Fang Y., Yao C., Wang Y., Zhang Z., Wang X. (2022). The synergistic effects of PRDX5 and Nrf2 on lung cancer progression and drug resistance under oxidative stress in the zebrafish models. Oncol. Res..

[49] Zhao Y., Chen S., Shen F., Long D., Yu T., Wu M. (2019). In vitro neutralization of autocrine IL-10 affects Op18/stathmin signaling in non-small cell lung cancer cells. Oncol. Rep..

[50] Li B., Nasser M.I., Masood M., Adlat S., Huang Y., Yang B. (2020). Efficiency of Traditional Chinese medicine targeting the Nrf2/HO-1 signaling pathway. Biomed. Pharmacother..

[51] Liu P., Wu D., Duan J., Xiao H., Zhou Y., Zhao L. (2020). NRF2 regulates the sensitivity of human NSCLC cells to cystine deprivation-induced ferroptosis via FOCAD-FAK signaling pathway. Redox Biol..

[52] Ni Y., Liu J., Zeng L., Yang Y., Liu L., Yao M. (2023). Natural product manoalide promotes EGFR-TKI sensitivity of lung cancer cells by KRAS-ERK pathway and mitochondrial Ca2+ overload-induced ferroptosis. Front. Pharmacol..

[53] Lou J.-S., Zhao L.-P., Huang Z.-H., Chen X.-Y., Xu J.-T., Tai W.C.-S. (2021). Ginkgetin derived from Ginkgo biloba leaves enhances the therapeutic effect of cisplatin via ferroptosis-mediated disruption of the Nrf2/HO-1 axis in EGFR wild-type non-small-cell lung cancer. Phytomedicine.

[54] Feng S., Li Y., Huang H., Huang H., Duan Y., Yuan Z. (2023). Isoorientin reverses lung cancer drug resistance by promoting ferroptosis via the SIRT6/Nrf2/GPX4 signaling pathway. Eur. J. Pharmacol..

[55] Xu F., Zhang J., Ji L., Cui W., Cui J., Tang Z. (2023). Inhibition of non-small cell lung cancer by ferroptosis and apoptosis induction through P53 and GSK-3β/nrf2 signal pathways using qingrehuoxue formula. J. Cancer.

[56] Tolomeo M., Cascio A. (2021). The multifaced role of STAT3 in cancer and its implication for anticancer therapy. Int. J. Mol. Sci..

[57] Wang J.-G., Li D.-L., Fan R., Yan M.-J. (2023). Zerumbone combined with gefitinib alleviates lung cancer cell growth through the AKT/STAT3/SLC7A11 axis. Neoplasma.

[58] Batbold U., Liu J.-J. (2021). Artemisia santolinifolia-mediated chemosensitization via activation of distinct cell death modes and suppression of STAT3/survivin-signaling pathways in NSCLC. Molecules.

[59] Li F., Hao S., Gao J., Jiang P. (2023). EGCG alleviates obesity-exacerbated lung cancer progression by STAT1/SLC7A11 pathway and gut microbiota. J. Nutr. Biochem..

[60] Luo L., Xu G. (2022). Fascaplysin induces apoptosis and ferroptosis, and enhances anti-PD-1 immunotherapy in non-small cell lung cancer (NSCLC) by promoting PD-L1 expression. Int. J. Mol. Sci..

[61] Qian X., Zhu L., Xu M., Liu H., Yu X., Shao Q. (2023). Shikonin suppresses small cell lung cancer growth via inducing ATF3-mediated ferroptosis to promote ROS accumulation. Chem. Biol. Interact..

[62] Shan G., Minchao K., Jizhao W., Rui Z., Guangjian Z., Jin Z. (2023). Resveratrol improves the cytotoxic effect of CD8+ T cells in the tumor microenvironment by regulating HMMR/Ferroptosis in lung squamous cell carcinoma. J. Pharm. Biomed. Anal..

[63] Lai X., Shi Y., Zhou M. (2023). Dihydroartemisinin enhances gefitinib cytotoxicity against lung adenocarcinoma cells by inducing ROS‐dependent apoptosis and ferroptosis. Kaohsiung J. Med. Sci..

[64] Iida Y., Okamoto Κatsuyama M., Maruoka S., Mizumura K., Shimizu T., Shikano S. (2021). Effective ferroptotic small-cell lung cancer cell death from SLC7A11 inhibition by sulforaphane. Oncol. Lett..

[65] Li X., Zhu S., Li Z., Meng Y.Q., Huang S.J., Yu Q.Y. (2022). Melittin induces ferroptosis and ER stress-CHOP-mediated apoptosis in A549 cells. Free Radic. Res..

[66] Zhai F., Liang Q., Wu Y., Liu J., Liu J. (2022). Red ginseng polysaccharide exhibits anticancer activity through GPX4 downregulation-induced ferroptosis. Pharm. Biol..

[67] Zhang W., Jiang B., Liu Y., Xu L., Wan M. (2022). Bufotalin induces ferroptosis in non-small cell lung cancer cells by facilitating the ubiquitination and degradation of GPX4. Free Radic. Biol. Med..

[68] Wu C.-Y., Yang Y.-H., Lin Y.-S., Chang G.-H., Tsai M.-S., Hsu C.-M. (2021). Dihydroisotanshinone I induced ferroptosis and apoptosis of lung cancer cells. Biomed. Pharmacother..

[69] Gao M., Lai K., Deng Y., Lu Z., Song C., Wang W. (2023). Eriocitrin inhibits epithelial-mesenchymal transformation (EMT) in lung adenocarcinoma cells via triggering ferroptosis. Aging (Albany NY).

[70] Cai S., Ding Z., Liu X., Zeng J. (2023). Trabectedin induces ferroptosis via regulation of HIF-1α/IRP1/TFR1 and Keap1/Nrf2/GPX4 axis in non-small cell lung cancer cells. Chem. Biol. Interact..

[71] Shao M., Jiang Q., Shen C., Liu Z., Qiu L. (2022). Sinapine induced ferroptosis in non-small cell lung cancer cells by upregulating transferrin/transferrin receptor and downregulating SLC7A11. Gene.

[72] Wu L., Xu G., Li N., Zhu L., Shao G. (2023). Curcumin analog, HO-3867, induces both apoptosis and ferroptosis via multiple mechanisms in NSCLC cells with wild-type p53. Evidence-Based Complement Altern Med.

[73] Rah B., Shafarin J., Hamad M., Muhammad J.S. (2023). Sclareol induces cell cycle arrest and ROS‐mediated apoptosis and ferroptosis in lung adenocarcinoma cells. J. Biochem. Mol. Toxicol..

[74] Zhang R., Pan T., Xiang Y., Zhang M., Xie H., Liang Z. (2022). Curcumenol triggered ferroptosis in lung cancer cells via lncRNA H19/miR-19b-3p/FTH1 axis. Bioact. Mater..

[75] Tsai Y., Xia C., Sun Z. (2020). The inhibitory effect of 6-gingerol on ubiquitin-specific peptidase 14 enhances autophagy-dependent ferroptosis and anti-tumor in vivo and in vitro. Front. Pharmacol..

[76] Yin Q., Ping L., Sheng H., Chang J., Li W., Lv S. (2023). Pseudolaric acid B triggers ferritinophagy and ferroptosis via upregulating NCOA4 in lung adenocarcinoma cells. J. Cancer Res. Therapeut..

[77] Li J., Yuan J., Li Y., Wang J., Xie Q., Ma R. (2022). d-Borneol enhances cisplatin sensitivity via autophagy dependent EMT signaling and NCOA4-mediated ferritinophagy. Phytomedicine.

[78] Tang X., Ding H., Liang M., Chen X., Yan Y., Wan N. (2021). Curcumin induces ferroptosis in non‐small‐cell lung cancer via activating autophagy. Thorac Cancer.

[79] Shen H., Wei Y., Yang Q., Cai Y., Zhu K., Chen X. (2023). Scoparone induces both apoptosis and ferroptosis via multiple mechanisms in non-small-cell lung cancer cells. Toxicol. Vitro.

[80] Huang F., Pang J., Xu L., Niu W., Zhang Y., Li S. (2022). Hedyotis diffusa injection induces ferroptosis via the Bax/Bcl2/VDAC2/3 axis in lung adenocarcinoma. Phytomedicine.

[81] Chen P., Wu Q., Feng J., Yan L., Sun Y., Liu S. (2020). Erianin, a novel dibenzyl compound in Dendrobium extract, inhibits lung cancer cell growth and migration via calcium/calmodulin-dependent ferroptosis. Signal Transduct. Targeted Ther..

[82] Kang M.-J., Ryu H.W., Oh E.S., Song Y.N., Huh Y.H., Park J.-Y. (2023). Diplacone isolated from Paulownia tomentosa mature fruit induces ferroptosis-mediated cell death through mitochondrial Ca2+ influx and mitochondrial permeability transition. Int. J. Mol. Sci..

[83] Chen X., Yu C., Kang R., Tang D. (2020). Iron metabolism in ferroptosis. Front. Cell Dev. Biol..

[84] Feng H., Schorpp K., Jin J., Yozwiak C.E., Hoffstrom B.G., Decker A.M. (2020). Transferrin receptor is a specific ferroptosis marker. Cell Rep..

[85] Chen S., Zhao Y., Shen F., Long D., Yu T., Lin X. (2019). Introduction of exogenous wild-type p53 mediates the regulation of oncoprotein 18/stathmin signaling via nuclear factor-κB in non-small cell lung cancer NCI-H1299 cells. Oncol. Rep..

[86] Zhang X.-D., Liu Z.-Y., Wang M.-S., Guo Y.-X., Wang X.-K., Luo K. (2023). Mechanisms and regulations of ferroptosis. Front. Immunol..

[87] Shindo M., Torimoto Y., Saito H., Motomura W., Ikuta K., Sato K. (2006). Functional role of DMT1 in transferrin-independent iron uptake by human hepatocyte and hepatocellular carcinoma cell, HLF. Hepatol. Res..

[88] Tang Z., Jiang W., Mao M., Zhao J., Chen J., Cheng N. (2021). Deubiquitinase USP35 modulates ferroptosis in lung cancer via targeting ferroportin. Clin. Transl. Med..

[89] Hu W., Zhou C., Jing Q., Li Y., Yang J., Yang C. (2021). FTH promotes the proliferation and renders the HCC cells specifically resist to ferroptosis by maintaining iron homeostasis. Cancer Cell Int..

[90] Quiles del Rey M., Mancias J.D. (2019). NCOA4-mediated ferritinophagy: a potential link to neurodegeneration. Front. Neurosci..

[91] Pope L.E., Dixon S.J. (2023). Regulation of ferroptosis by lipid metabolism. Trends Cell Biol..

[92] Yang Y., Zhu T., Wang X., Xiong F., Hu Z., Qiao X. (2022). ACSL3 and ACSL4, distinct roles in ferroptosis and cancers. Cancers.

[93] Lu L., Zhai X., Li X., Wang S., Zhang L., Wang L. (2022). Met1-specific motifs conserved in OTUB subfamily of green plants enable rice OTUB1 to hydrolyse Met1 ubiquitin chains. Nat. Commun..

[94] Guo J., Zhou Y., Liu D., Wang M., Wu Y., Tang D. (2022). Mitochondria as multifaceted regulators of ferroptosis. Life Metab.

[95] He Y., Wang W., Yang T., Thomas E.R., Dai R., Li X. (2022). The potential role of voltage-dependent anion channel in the treatment of Parkinson's disease. Oxid. Med. Cell. Longev..

[96] Zhang Q., Yi H., Yao H., Lu L., He G., Wu M. (2021). Artemisinin derivatives inhibit non-small cell lung cancer cells through induction of ROS-dependent apoptosis/ferroptosis. J. Cancer.

[97] Ke K., Li L., Lu C., Zhu Q., Wang Y., Mou Y. (2022). The crosstalk effect between ferrous and other ions metabolism in ferroptosis for therapy of cancer. Front. Oncol..

[98] Xu L., Huang X., Lou Y., Xie W., Zhao H. (2022). Regulation of apoptosis, autophagy and ferroptosis by non-coding RNAs in metastatic non-small cell lung cancer. Exp. Ther. Med..

[99] Huang J., Deng C., Guo T., Chen X., Chen P., Du S. (2023). Cinobufotalin induces ferroptosis to suppress lung cancer cell growth by lncRNA LINC00597/hsa-miR-367-3p/TFRC pathway via resibufogenin. Anti Cancer Agents Med. Chem..

[100] Fu F., Wang W., Wu L., Wang W., Huang Z., Huang Y. (2023). Inhalable biomineralized liposomes for cyclic Ca(2+)-burst-centered endoplasmic reticulum stress enhanced lung cancer ferroptosis therapy. ACS Nano.

[101] Zhou Q., Meng Y., Li D., Yao L., Le J., Liu Y. (2024). Ferroptosis in cancer: from molecular mechanisms to therapeutic strategies. Signal Transduct. Targeted Ther..

[102] Chen X., Liao Y., Long D., Yu T., Shen F., Lin X. (2017). The Cdc2/Cdk1 inhibitor, purvalanol A, enhances the cytotoxic effects of taxol through Op18/stathmin in non-small cell lung cancer cells in vitro. Int. J. Mol. Med..

[103] Han N., Yang Z.-Y., Xie Z.-X., Xu H.-Z., Yu T.-T., Li Q.-R. (2023). Dihydroartemisinin elicits immunogenic death through ferroptosis-triggered ER stress and DNA damage for lung cancer immunotherapy. Phytomedicine.

